# A 3D Printed Ready-Mixed Concrete Power Distribution Substation: Materials and Construction Technology

**DOI:** 10.3390/ma12091540

**Published:** 2019-05-10

**Authors:** Guangchao Ji, Tao Ding, Jianzhuang Xiao, Shupeng Du, Jun Li, Zhenhua Duan

**Affiliations:** 1 Department of Structural Engineering, College of Civil Engineering, Tongji University, Shanghai 200092, China; 15241770066@163.com (G.J.); tding@tongji.edu.cn (T.D.); zhduan@tongji.edu.cn (Z.D.); 2 Liaoning Green Printing Technology Co., Ltd., Yingkou City 115214, China; gelinpu3d@163.com; 3 College of Civil Engineering and Architecture, Dalian University, Liaoning 116622, China; lijun47193@126.com

**Keywords:** 3D printing, ready-mixed concrete, equipment system, coarse aggregate, construction

## Abstract

Currently, 3D concrete printing technology is not yet able to print ready-mixed concrete with coarse aggregates. Based on an independently developed 3D printing construction equipment system and optimized concrete materials, a 3D concrete printer that can directly print ready-mixed concrete is developed. This paper introduces the whole 3D printing process for one power distribution substation in detail, including the printing equipment, key software, concrete preparation, printing process, and construction inspection. This investigation will provide valuable design and construction experience for the future construction of 3D concrete printing.

## 1. Introduction 

Architectural 3D printing is an additive-based molding technology based on a computer digital model. It can be quickly formed by printing building materials layer by layer, thus achieving the advantages of high efficiency, low cost, and environmental protection.

In 1997, Pegna [1] proposed to apply the additive manufacturing process to automatic construction to replace the complex assembly of structural members, and build a masonry structure by using sand and cement as materials. It is the origin of architectural 3D printing. In 1998, Khoshnevis and Dutton [2] developed a contour crafting designed for architectural 3D printing, which uses a computer-controlled trowel to create a smooth and precise structural surface with high building surface quality and fast construction speed. In addition, the possibility of using the technology to print buildings on the moon has been imagined. In 2000, Benhabib et al. [3] put forward an improved hybrid rule model to predict the mechanical properties of short glass fiber reinforced printing inks. Experiments were carried out to verify the effectiveness of empirical data based on fiber model interfaces, fiber geometry, and length distribution. In 2002, Kwon et al. [4] conducted an experimental and simple finite element simulation study on the material flow pattern during extrusion and deposition in the contour printing process based on ceramic materials (such as clay), and they concluded that the square port can better realize the fusion between printing layers and the ideal external surface contour. In 2007, Bosscher et al. [5] of Ohio University in the United States proposed a contour process improvement system consisting of a translational suspension robot, which used a steel frame as a skeleton to control the three-dimensional movement of the terminal nozzle by cable to provide the possibility of 3D printing for larger-sized buildings. In 2007, Cesaretti et al. [6] developed a method by spraying a binder to glue-hardening layer-by-layer gravel powder for stack forming, namely, D-Shape printing technology. In 2011, Le et al. [7] discussed the hardened properties of a nozzle-extruded fiber-reinforced high-performance concrete and conducted experiments to examine the influence of different mixes for fresh properties (printability, workability, setting time, etc.) and hardened properties (density, compressive strength, interlaminar bond strength, and drying shrinkage). In 2016, Gosselin et al. [8] studied how to use 3D printing technology to print 3D large complex graphics instead of 2.5D (vertical extension of planar shape) and pointed out the advantages of 3D printing technology in building complex three-dimensional structures.

In 2017, Panda et al. [9] studied the effects of different lengths and amounts of glass fiber geopolymer mortar on the strength of 3D printed materials, and pointed out that the mechanical properties of printed samples are obviously related to the loading direction. Zareiyan and Khoshnevis [10] studied the effect of cross-linking structure on the bond strength between 3D printing layers, and indicated that the bond strength varies greatly with the variation of cross-linking structure. Khalil et al. [11] used a combination of sulphoaluminate cement and ordinary Portland cement to prepare a new material suitable for 3D printing. It was found that the incorporation of sulphoaluminate cement significantly improved the constructability of the material. The test analysis on hydration heat and compressive strength demonstrated that the properties were not much different. Kazemian et al. [12] studied the cement-based materials doped with silica fume and nanoclay from the aspects of print quality, stability, and setting time, and concluded that the incorporation of silica fume and nanoclay can significantly improve the shape stability. Hambach and Volkmer [13] studied Portland cement 3D printing material incorporating reinforced short fibers. The compressive strength of the material could reach 80 MPa, and the possibility of controlling the direction of the printing path as a means to control fiber orientation within the printed structures was also discussed.

In 2018, Soltan and Li [14] conducted a performance test of a cement-based material incorporating short polymer fibers, and analyzed the influence of composition and process parameters on its fluidity. Wolfs et al. [15] established a numerical model for analyzing the performance of fresh 3D printed concrete, analyzed the elastic modulus, cohesion, compressive strength, and shear strength of the material through experiments, and verified the correctness of the model. Sanjayan et al. [16] and Paul et al. [17] conducted compressive, flexural, and interlaminar strength tests on 3D printed concrete materials, and the results showed that the compressive and flexural strength of the materials depended on the test direction. Panda et al. [18] analyzed the relationship between the interlaminar tensile bond strength and the time interval of printing layers, the nozzle speed and the nozzle height. Ma et al. [19] proposed an environmentally friendly cementitious mixture that is compatible with an extrusion-based printing process. Printable properties of mixtures with six substitute ratios of tailing to sand were investigated.

However, at present, architectural 3D printing technology is still in the research and development stage. From the perspective of application, there are mainly three technical difficulties. First, at the material level, traditional building materials are basically composed of inorganic materials, which makes the molding time very long and makes it difficult to satisfy the requirements of the 3D printing process. It is impossible to change the material properties by hot melt of printing materials such as metal powder and plastic. Second, some building materials that satisfy the requirements of 3D printing technology, such as certain special mortars and special polymers, are difficult to meet the material and structural requirements of buildings, and are also not common building materials. Finally, in the field of equipment application, some institutions and companies have introduced 3D printing components in the factory, and then transport the components to the site for building assembly. This method is more similar to a prefabricated building, and the transportation radius is limited, which does not take advantage of the high efficiency and low cost of building 3D printing. These technical difficulties lead directly to the fact that there are still no proven and fully promoted architectural 3D printing products and technologies in the market.

This paper proposes a 3D printing technology and an overall solution from design to on-site, which can directly print commercial ready-mixed concrete. In-filed printing of a power distribution substation will be introduced in detail. Since the traditional commercial ready-mixed concrete is adopted as the printing material, this solves not only the application problem of building materials, but also the structural design for the buildings of this project.

## 2. Printer Equipment and Materials

### 2.1. 3D Printing Equipment

At present, various kinds of 3D printers such as polypropylene fiber concrete printers [7,20], nylon fiber concrete printers, binder gelled grit powder printers [21], and gypsum material printers [22] have been proposed. However, from quantity production to practical application, much technical research work should still be done if the new building materials printed by these 3D printers are to be applied. Therefore, research and development of a 3D printer that can directly print commercial ready-mixed concrete has become an urgent need.

**Figure 1:**
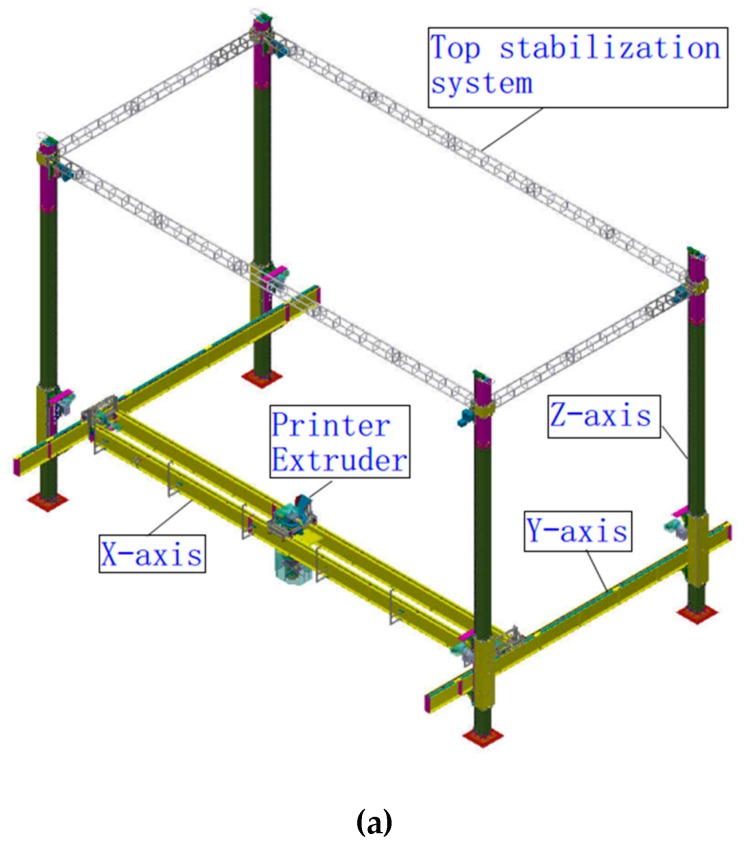

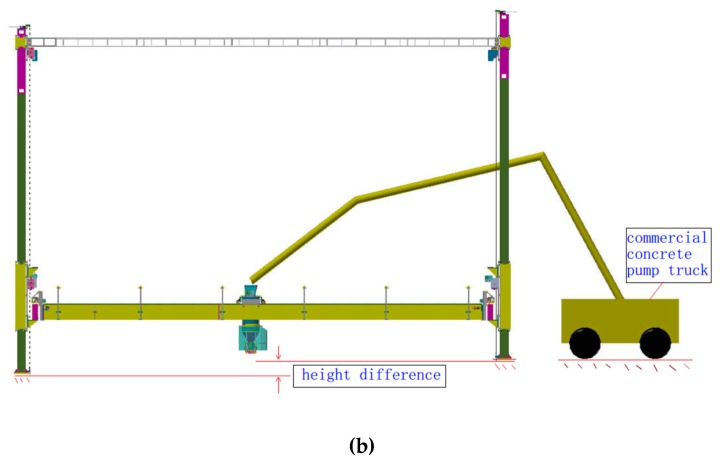
Column-type 3D printer for ready-mixed concrete: (**a**) Axonometric view and (**b**) Elevation view.

The 3D printer proposed in this study is a column-type mechanical structure, and a four-column frame is set up to implement 3D printing in the frame. As shown in Figure 1a,b, the 3D printer is divided into several structural parts: *X*-axis track, *Y*-axis track, *Z*-axis column track, print head, top stabilization system, and ready-mixed concrete pump truck. The *X*-axis and *Y*-axis tracks can control the horizontal movement of the print head, while the horizontal system *X*-axis and *Y*-axis can be vertically raised by the trolley on the *Z*-axis, which satisfies the movement of the print head in the *X*-, *Y*-, and *Z*-axes. The column-type 3D printer has an *X*-axis width and a *Z*-axis height of 20 m. It can print buildings with a width and height of no more than 18 m. The length of the *Y*-axis can be extended indefinitely by the increase in the number of Z-axis columns. Therefore, the column-type concrete 3D printer is simple to install and can print concrete structures of less than six stories. Moreover, the print head and arm of the concrete pump truck are driven independently and each equipped with a positioning sensor that detects the relative position for each other. When the concrete in the print head is continuously printed, the arm of the concrete pump truck itself can perform positioning and feeding work independently.

The column-type mechanical structure provides a printing platform for 3D printers, and the main difficulty lies in how to develop a printer suitable for printing ready-mixed concrete. The material composition of ready-mixed concrete is sand, coarse aggregate, cement, and water. The existence of coarse aggregates in ready-mixed concrete poses a huge challenge to the development of 3D printing equipment. The main reason is that it is difficult to develop a print head that can print concrete containing coarse aggregate.

**Figure 2:**
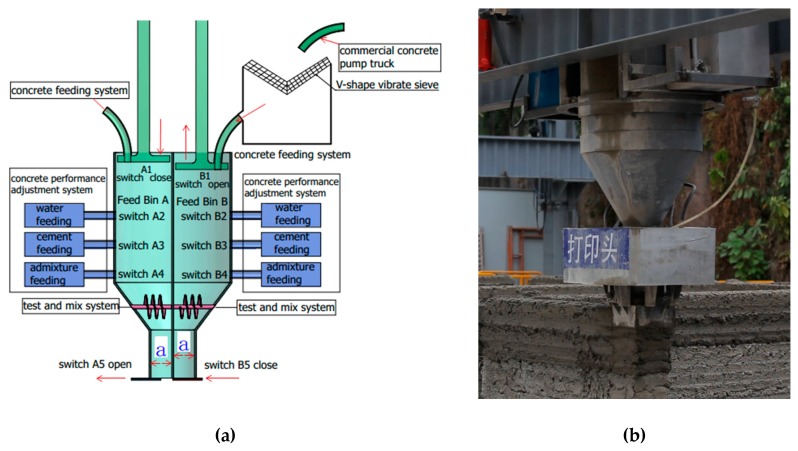
Schematic of double-assisted print head (**a**) Design diagram and (**b**) Print head in the field

The architectural 3D printing system introduced in this study is a commercial ready-mixed concrete building 3D printing system independently developed by the authors. The printing system is characterized in that it comprises a double-assisted print head, which can continuously print uninterrupted ready-mixed concrete with aggregate less than 15 mm in diameter. Figure 2 shows a schematic of the double-assisted print head. It is divided into two feed bins with four systems: reciprocating plugging power system, concrete feeding system, concrete performance test and mix system, and concrete performance adjustment system.

The working principle of the double-assisted print head is that feed bins A and B can work together at the same time. When one feed bin is extruding and printing concrete, the concrete feeding and slump automatic adjustment starts to work in the other feed bin, in order to achieve the uninterrupted printing of concrete.

The reciprocating plugging power system is composed of pistons A and B, as seen in Figure 2. When feed bin A is printing and the concrete is feeding into feed bin B, piston C moves downward and switch A5 is opened. The concrete in feed bin A moves downward by piston A’s squeezing to obtain the concrete printing, and the computer controls the concrete printing speed by controlling the descending speed of piston A. At the same time, piston D moves up to the top and closes switch B5, and opens the concrete feeding system for concrete feeding into feed bin B.

The function of the concrete feeding system is to transport the concrete from the mixing equipment into the feeding bin of the print head through the pipeline and the screw power, and to start and close the feeding by cooperating with switches A1 and B1. 

**Figure 3:**
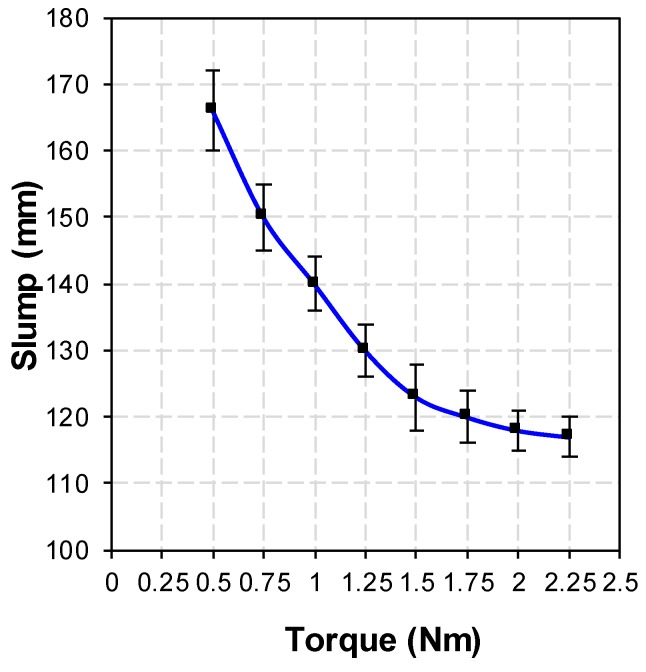
Relationship between torque and slump for the 3D concrete printing system.

It is known that the transportation and feeding processes will introduce uncertain changes to the concrete working performance. As a result, the workability test and adjustment in the feeding bin become important to the concrete printing. The concrete performance test and mix system is equipped with a servo motor that can perform the torque test. As shown in Figure 2, the spiral blades in feed bins A and B are driven by the servo motor, respectively. The rotation of the spiral blades feeds back the magnitude of the servo motor torque. Through repeated tests, the relationship between torque and slump is shown in Figure 3, when the diameter spiral blade is 50 mm, the pitch is 25 mm, and the screw is 120 mm.

When the concrete slump in the feed bin is between 120 and 130 mm, the printing effect will be the best, that is, the servo motor torque is between 1.25 and 1.75 Nm. If the motor torque is not within this range, the concrete performance adjustment system will be activated. The workability of concrete will be adjusted by adding water, superplasticizer, additives, etc. At the same time, the detection system feeds back the torque data every second. When the torque reaches between 1.25 and 1.75 Nm, the performance adjustment system will be turned off to start printing.

It is important to note that the ready-mixed concrete 3D printing system described in this study is equipped with a screw for torque testing in the concrete performance testing system. Therefore, the diameter of coarse aggregate in the concrete is required to be no larger than 15 mm. A V-shape vibrating sieve is designed at the upper end of the concrete feeding system. This sieve has a pore spacing of 15 mm and can filter out aggregates larger than 15 mm in diameter. In addition, if the concrete in the print head is continuously vibrated during the printing process, the mechanical system error caused by the vibration process will be definitely unacceptable. However, it is usually difficult for the printed concrete without any treatment to meet the strength requirement. The squeeze 3D printing system can make the concrete compact by pressure, so that the concrete strength can meet the requirements. 

**Figure 4:**
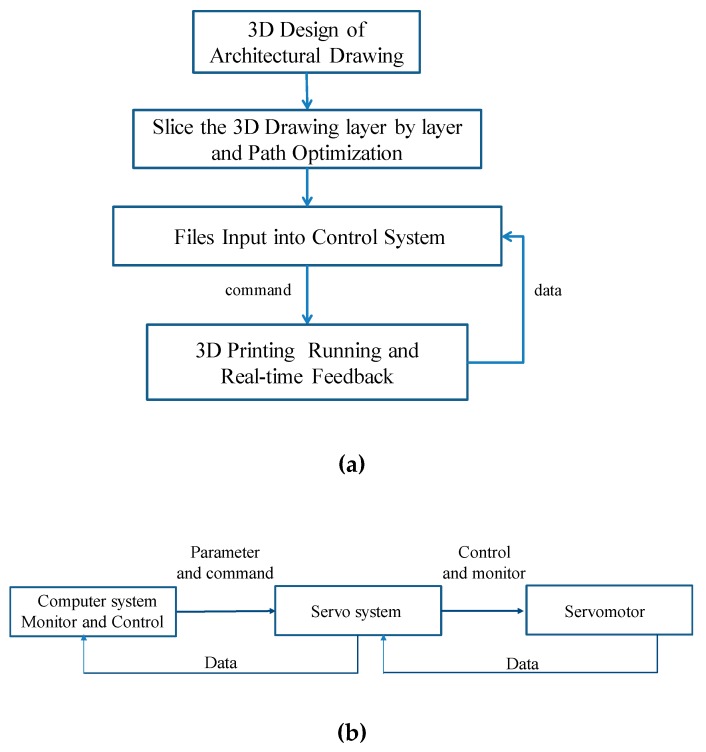
The software system (**a**) Running process for the software and (**b**) feedback control system.

**Figure 5:**
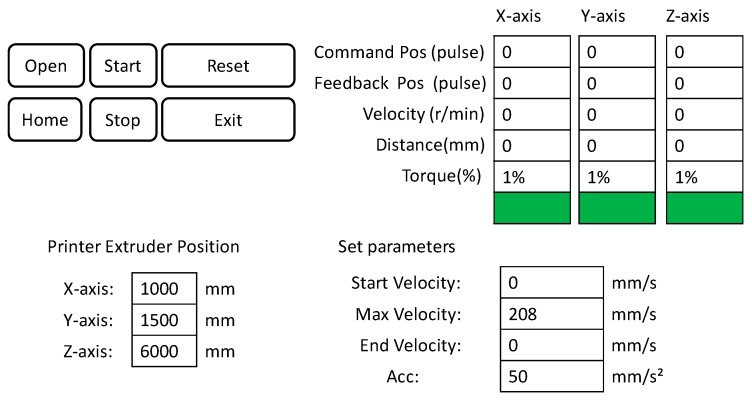
Control interface of the operating device.

The running process for the software system of this 3D printer is shown in Figure 4a and the servo feedback system is adopted into the software control, as shown in Figure 4b. The characteristics of this software system are as follows. First, the real-time feedback of the coordinate information solves the step-out phenomenon caused by the frequent high-speed start and stop of the motor and helps the whole system operate accurately. Second, the feedback data displays parameters such as motor load, motor temperature, and printing speed. Once motor overload occurs, the motor temperature is too high or even short-circuited, printing work stops immediately and the power supply is disconnected to ensure safety, Third, the 3D printing operation parameters are set manually, such as maximum running speed, acceleration, speed of print head, etc. The maximum speed of the system is within 0.5 m/s, and the optimal range of acceleration is between 0.05 m/s² and 0.2 m/s². Finally, it has a storage function at breakpoint. When an unexpected situation occurs, such as a power failure, the servo system immediately stores the current coordinate position. Later printing at breakpoint can be achieved when the system is restarted. Figure 5 shows the control interface of the operating device.

### 2.2. Print Concrete Materials

The most critical properties for the printing concrete in the fresh state are extrudability and buildability. Extrudability relates to the ability of delivering fresh concrete through a hopper and pumping system to a nozzle where the concrete must be extruded as a continuous filament. It is mainly affected by the workability. Moreover, the printed concrete filaments should be formed with minimal deformation under the weight of subsequent layers. Additionally, the lower filaments should bond to the upper ones to build monolithic components. Therefore, this printing ready-mixed concrete requires a buildability which relates to the ability of printing a certain number of layers or height. Buildability also depends on the workability and mix proportions.

In this study, a 3D printer was used to optimize the workability range of concrete which was ready to be extruded. It is known that higher slump has a negative effect on the deformation of concrete after extrusion, whereas lower slump may cause the noncontinuous flow of concrete which affects the buildability and hardened properties of concrete. After a series of trials, a slump of 110 mm was recommended as the most suitable workability in application. 

After several trials, the mix ratio of 3D printing concrete was optimized and is shown in Table 1. PO 42.5 cement was used for the concrete. The fine aggregate used was sand with a fineness modulus of 2.8, the coarse aggregate had a particle size of 5–15 mm, and the admixture used was fly ash. The amount of accelerator was about 3–5% to the total amount of cement in the concrete. The printed concrete reached the initial setting in 5–10 minutes. The optimized 3D printing concrete material enabled continuous printing without interruption.

**Figure 6:**
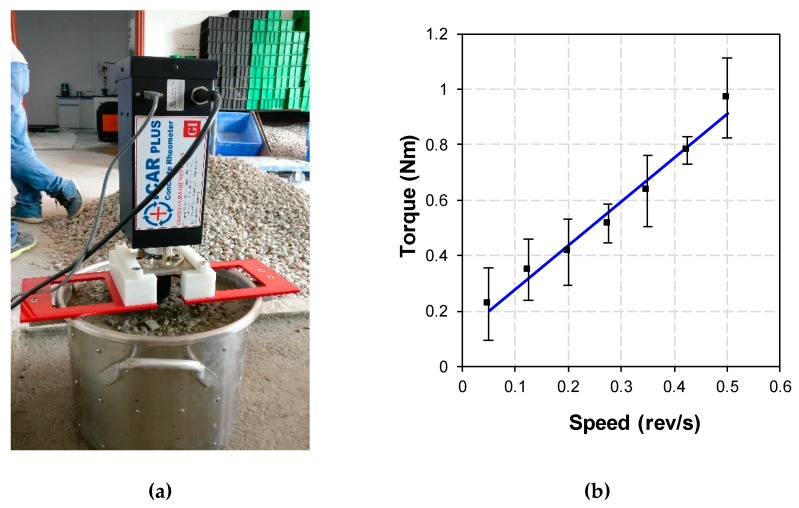
Rheological test of print ready-mixed concrete. (**a**) ICAR rheometer and (**b**) Relationship between torque and rotational velocity.

The rheological parameters of the concrete were tested in this study using an International Center for Aggregate Research (ICAR) rheometer (Shanghai LREL Instruments. CO., LTD, Denmark ), as shown in Figure 6a, with a four-bladed vane. The flow curve test worked within seven velocity stages and every stage maintained a certain time to reach static state. Using the flow curve test, the relationship between torque and rotational velocity was obtained (Figure 6b). By using the Reiner–Riwlin equation and the Bingham model [23], the dynamic yield stress and plastic viscosity were obtained. The results showed that the rheological parameters satisfied the requirements of pumpability and extrudability.

Based on the above mixing ratio for the printed concrete, concrete was cast into a cube test mold. The Chinese standard for the test method of mechanical properties on ordinary concrete (GB/T50081-2002) [24] was adopted, and a hydraulic compression testing machine (Jinan Chuanbai Equipment, Jinan, Shandong, China) was used to evaluate the compressive strength of the material. The printed concrete and the common ready-mixed concrete results obtained after 28 days are shown in Table 2. It can be observed that the strength of the printed concrete reduced by 2–4 MPa compared to the common ready-mixed concrete. It is indicated that the addition of an accelerator as the admixture could decrease the mechanical strength of the print concrete. The accelerator accelerates the hydration of the silicate minerals C3S, C2S, and C4AF and also introduces cracks and voids inside the concrete, which will result in strength loss for the concrete.

## 3. Distribution Substation Project

### 3.1. Description

**Figure 7:**
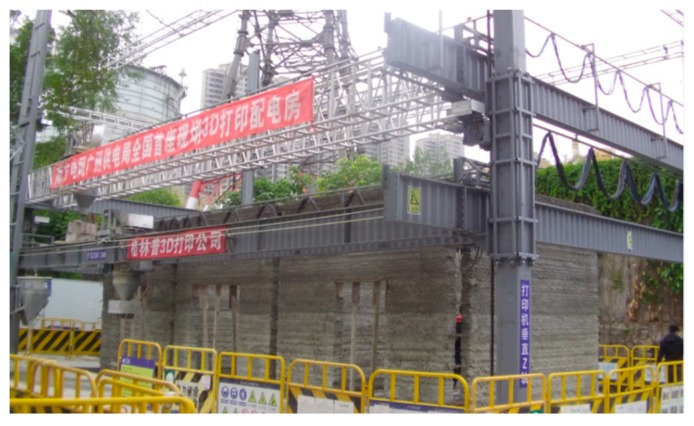
Overview of the 3D printer.

**Figure 8:**
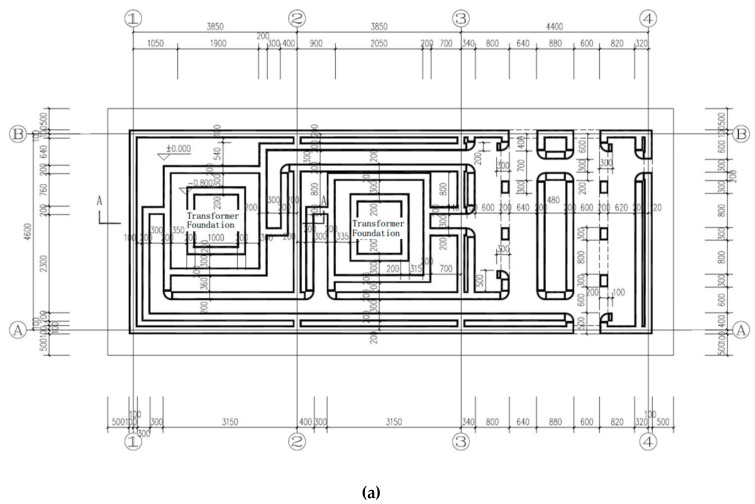

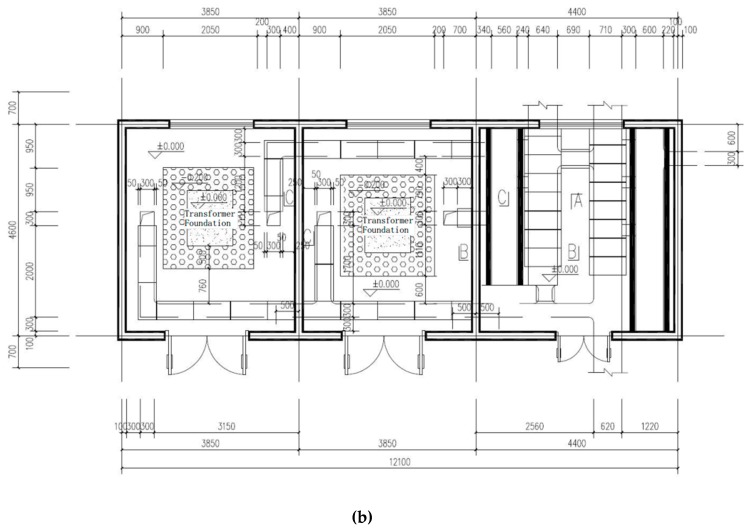
Plan view of 3D printed power distribution substation (**a**) Plan view of cable trench and (**b**) Plan view of cable trench.

With the techniques in equipment and materials for 3D printing mentioned above, this paper introduces the world’s first application of a 3D printed power distribution substation by adopting commercial ready-mixed concrete in China. The full picture of the printer is shown in Figure 7. Figure 8 shows the design of the power distribution substation. It can be seen that the power distribution substation has a length of 12.1 m, a width of 4.6 m, and a total height of 4.6 m (the part below the ground is 0.5 m, and the part above the ground is 4.1 m).

### 3.2. Design and 3D Printing

According to the design, the concrete strength of the wall should not be less than 20 MPa. The wall part of the power distribution substation is printed with C25 ready-mixed concrete, with a diameter of the coarse aggregate less than 15 mm. During the printing process of the power distribution substation, the actual strength of the wall concrete is higher than 20 MPa, which meets the structural design requirements.

**Figure 9:**
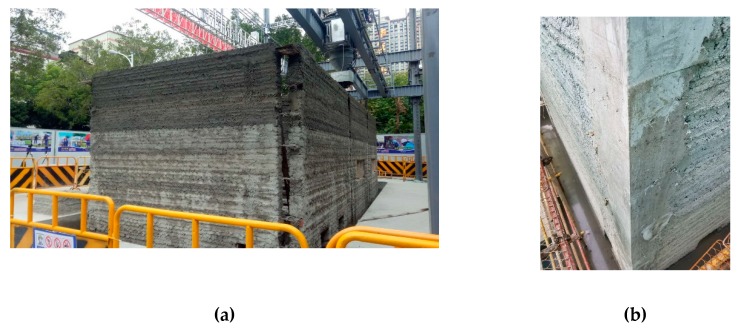
Constructional columns (**a**) Reserved position for constructional columns during printing and (**b**) Constructional columns after casting.

**Figure 10:**
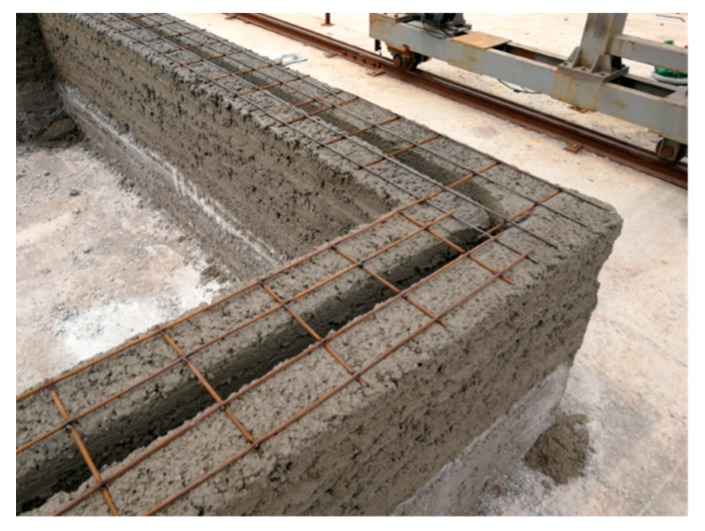
Horizontal steel mesh.

Constructional columns and ring beams were employed in the structural design of the project to meet the seismic requirements. Therefore, the project optimizes the path of the concrete printing process. The concrete wall is printed horizontally at first, and the position of constructional columns is reserved during the printing process. After the concrete printing wall is finished, constructional columns are then built by means of supporting formwork, tying steel bars and casting concrete, as seen in Figure 9. In addition, the project combines the characteristics of 3D printing, and a horizontal steel mesh is placed at a height of 500 mm intervals to connect with the constructional column, as shown in Figure 10. Horizontal steel mesh and printed concrete were both adopted to build the lintels and ring beams, in order to enhance the integrity of the overall structure.

It is worth noting that for the printing of the lintel above the door and window holes, it is still necessary to use the traditional method of constructing the formwork, that is, using the prefabricated wooden formwork to support the lintel. The length of the wooden formwork is the same as the length of the lintel, whereas the width is slightly larger than the lintel width. Using the 3D printing method on the wooden mold support, the construction of the lintel is achieved.

During the construction, the 3D design model is first carried out, and then the 3D printing slice technology is used to slice the building model, generating a layer of digitized code. In addition, using the real-time detection and adjustment function of the 3D printing system, the printing speed, the direction of the squeegee, the motor state, and the system temperature are detected and adjusted.

**Figure 11:**
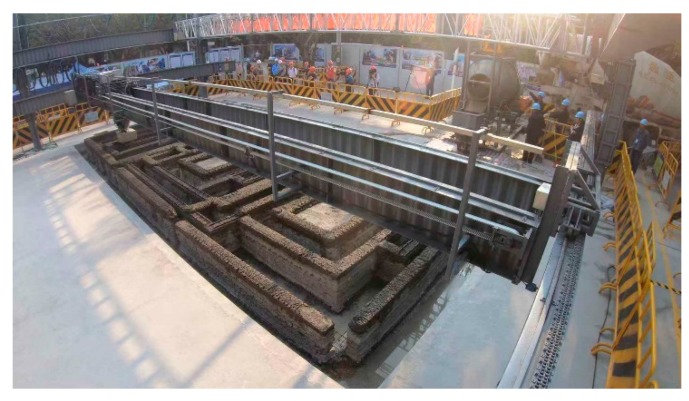
3D printing of cable trench, foundation, and oil collection tank below the ground.

**Figure 12:**
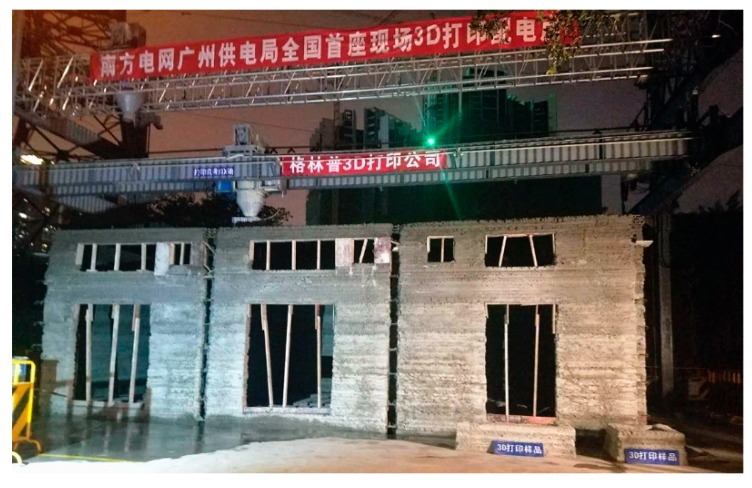
Power distribution substation after 3D printing.

In the 3D printing design of the cable trench, equipment foundation, and oil collection tank below the ground, the extension length of the print head was optimized, and the complex underground structure was printed, as shown in Figure 11. Figure 12 demonstrates the power distribution substation after 3D printing. The Schmidt hammer rebound test was developed to determine the compressive strength of concrete, and has been used to determine the hardness and compressive strength of concrete [25]. The rebound hammer test is a non-destructive testing method and can be applied both in the die laboratory and in the field. At least 20 individual tests were conducted on the printed buildings, in which the longitudinal axis of the hammer was perpendicular to the face plane. Corresponding uniaxial compressive strength values were calculated. Rebound tests were performed 28 days after completing the printing of the power distribution substation, and the test results at multiple points were higher than 20 MPa. The 3D printing method, instead of the traditional artificial construction method, can carry out the complicated printing of cable trenches, oil collecting pools, and power equipment foundations at one time, thereby achieving high efficiency, low labor, low cost, and benefits to the environment.

## 4. Discussion 

The main advantage of this 3D printing power distribution substation project is the use of commercial ready-mixed concrete as the printing material, thus meeting the requirements of current regulations in materials and structural design. In addition, it is a full-scale concrete building printed by a 3D printer, which is also rare in the world at present.

The total construction time of the power distribution substation project using traditional cast-in-place concrete is 54 days, whereas the construction time using 3D printing is less than 35 days, time which is shortened by 30%. The number of working days for the main part of the building using 3D printing is only four days, which is a 60–80% reduction compared with the traditional construction method. Moreover, since 3D printing replaces the traditional construction process, the dust pollution on the site is reduced and ensures a clean and tidy construction site. The construction waste is less than one ton, and the amount of waste generated is reduced by more than 60% in the construction field, which is highly beneficial to the environment.

The 3D printing of the building described in this paper uses coarse aggregate with a particle size of 5–15 mm. Compared with the traditional mortar ink used in 3D printing concrete, it is a big step forward. In addition, a power distribution substation was successfully printed, which met the building standards of most countries in terms of structural design and material specifications. However, currently, the construction of 3D printing equipment can only be operated by a single print head. It is difficult to achieve the collaborative work of multi-printing heads simultaneously. If this can be realized in the future, the printing efficiency will be further increased. Moreover, the current print head can only print materials with a diameter of no more than 15 mm, and the degree of cooperation between the print head and the commercial ready-mixed concrete can be further improved. 

Additionally, although the highly automated 3D printer can replace manual work in a low-temperature state, due to the special nature of the hydration heat reaction of concrete, the concrete material that can be printed at a low temperature has not yet been formulated. Therefore, it is impossible to carry out 3D printing concrete construction in winter, especially in cold regions. Moreover, it should also be noted that construction with concrete printing not only reduces the labor needed, but can also increase the cost of materials, since a relatively higher-class concrete should be used for the practical engineering based on current technology. In terms of design and construction, the 3D printing construction of multi-story buildings must be improved and verified, such as columns that can be assembled using precast elements, which may highly improve the construction efficiency. At present, the bearing capacity of 3D printing concrete components or structures cannot be evaluated. In the future, it will be necessary to establish a structural performance evaluation system for 3D printing concrete buildings.

## 5. Conclusions

This paper introduces the whole 3D printing process of a power distribution substation completed in Guangzhou, China. The construction period of the project is short and the manual requirements are greatly reduced. Based on the self-developed printer, C25 ready-mixed concrete with 5–15 mm coarse aggregate was adopted as the printing material to construct the power distribution substation.

In terms of equipment, with a double-assisted printhead, it is possible to continuously print uninterrupted ready-mixed concrete during the printing process. In addition, the squeeze pressurization method replacing the vibrating system makes the compactness and strength of the concrete meet the material requirements. In terms of materials, for the printability and buildability of concrete, the 3D printing system is optimized which can meet the printing properties of ready-mixed concrete. Currently, construction with concrete printing reduces the labor needed, but can increase the cost of materials, since a relatively higher-class concrete should be used.

This project will provide valuable design and construction experiences for the future construction of concrete 3D printing, which has a certain guiding significance.

## Figures and Tables

**Table 1:** 3D printed ready-mixed concrete mix ratio.

Design Strength	Cement	Sand	Aggregate	Water	Admixture
C20	1	3.20	3.62	0.66	0.024
C25	1	2.98	3.40	0.60	0.032

**Table 2:** Comparison of concrete compressive strength tests.

Specimen	Concrete Design Strength (MPa)	Dimension (mm)	Compressive Strength (MPa)	Standard Deviations (MPa)
Printed	20 MPa	150 × 150 × 150	19.1	0.25
Normal	20 MPa	150 × 150 × 150	23.8	0.16
Printed	25 MPa	150 × 150 × 150	24.1	0.30
Normal	25 MPa	150 × 150 × 150	28.8	0.17
